# Very Early Involvement of Innate Immunity in Peripheral Nerve Degeneration in SOD1-G93A Mice

**DOI:** 10.3389/fimmu.2020.575792

**Published:** 2020-11-20

**Authors:** Daniela Francesca Angelini, Federica De Angelis, Valentina Vacca, Eleonora Piras, Chiara Parisi, Michele Nutini, Alida Spalloni, Francesca Pagano, Patrizia Longone, Luca Battistini, Flaminia Pavone, Sara Marinelli

**Affiliations:** ^1^ Neuroimmunology Unit, IRCCS Santa Lucia Foundation, Rome, Italy; ^2^ CNR—National Research Council, Institute of Biochemistry and Cell Biology, Rome, Italy

**Keywords:** peripheral nerve degeneration, demyelination, amyotrophic lateral sclerosis, mast cells, pro-inflammatory cytokine, autoimmunity, monocytes/macrophages, Wallerian-like degeneration

## Abstract

Recent preclinical and clinical evidence suggest that immune system has a role in the progression and prognosis of Amyotrophic Lateral Sclerosis (ALS), but the identification of a clear mechanism and immune players remains to be elucidated. Here, we have investigated, in 30 and 60 days (presymptomatic) and 120 days (symptomatic) old SOD1-G93A mice, systemic, peripheral, and central innate and adaptive immune and inflammatory response, correlating it with the progression of the neurodegeneration in neuromuscular junction, sciatic nerves, and spinal cord. Surprisingly, we found a very initial (45–60 days) presence of IgG in sciatic nerves together with a gradual enhancement of A20/TNFAIP3 (protein controlling NF-κB signalling) and a concomitantly significant increase and activation of circulating mast cells (MCs) as well as MCs and macrophages in sciatic nerve and an enhancement of IL-6 and IL-10. This immunological frame coincided with a myelin aggregation. The 30–60 days old SOD1-G93A mice didn’t show real elements of neuroinflammation and neurodegeneration in spinal cord. In 120 days old mice macrophages and monocytes are widely diffused in sciatic nerves, peripheral neurodegeneration reaches the tip, high circulating levels of TNFα and IL-2 were found and spinal cord exhibits clear signs of neural damage and infiltrating immune cells. Our results underpin a clear immunological disorder at the origin of ALS axonopathy, in which MCs are involved in the initiation and sustaining of inflammatory events. These data cannot be considered a mere epiphenomenon of motor neuron degeneration and reveal new potential selective immune targets in ALS therapy.

## Introduction

Amyotrophic Lateral Sclerosis (ALS) is a progressive neurological disorder, commonly defined as motor neurons disease because the most evident clinical signs are muscle weakness, loss of motor function, paralysis, and breathing problems due to implacable motor cells death. ALS cause and mechanism are still not completely clear. Currently, two opposing hypotheses exist (the dying forward and the dying back) (1) to explain the neuromuscular junction (NMJ) denervation. Post mortem autopsies have revealed, with the massive motor neuron death, the presence of proteins aggregates, intranuclear RNA deposits, astrogliosis and microgliosis (2). Muscle biopsies, acquired early during ALS development, have shown muscle denervation with scattered reinnervation, myopathic features, and chronic inflammation (3). Fischer and colleagues (4, 5) introduced how the distal axonopathy is the cause of the motor neuron degeneration in ALS. On the other hand, impairment of axons is recognized as origin of several human motor neuron pathologies (6) and it was observed that retrograde degeneration (from sciatic nerve toward spinal cord) induces apoptosis in motor neurons (7). Increased evidence indicates peripheral nervous system as first target in ALS disease and sciatic nerve the focus of initial abnormalities (8). Consolidated evidence (9, 10) in various animal models, demonstrated that neuroinflammation can be triggered as consequence of peripheral nerve degeneration; as a matter of fact, a great role in Wallerian degeneration is attributable to innate immune cells: macrophages and mast cells (MCs).

More recently, both clinical (11–14) and preclinical studies (15–18) have introduced the immune system as an additional active player, both centrally and peripherally (19) at different stages of the disease. Moreover, some clinical reports suggest a peculiar association of ALS with autoimmune disease such as myasthenia gravis (20–22). Autoimmunity, as a pathogenic mechanism in ALS, has been initially proposed in the nineties by Appel and colleagues (23, 24) who demonstrated in patients and mice the presence of specific autoantibodies against calcium channel. This hypothesis was not much examined in the following years and remained controversial even if numerous autoantibodies were recognized in ALS patients (25, 26). Some recent works, including clinical evidence (27), support the view that an immune reactivity against motor nerve terminals can lead to an alteration of calcium homeostasis. In particular, autoantibodies against P/Q calcium channels have been detected in SOD1-G93A mutant mice and humans, purposing an involvement in ALS development (28, 29).

P/Q calcium channels are highly expressed in NMJ and are well involved in neurotransmission. Mutations or dysfunctions in these channels (i.e. in the CaV2.1α1 subunit (P/Q), have been linked to heavy neuromuscular degenerative diseases such as the Lambert-Eaton myasthenia syndrome (30–32). The P/Q channels are present in axon-associated Schwann cells (SCs) (33) and related to myelin sheath formation and assembly. Furthermore, we have shown (34) that a reduction in P/Q calcium channels impairs muscular strength, as well as SCs proliferation and sciatic nerve remyelination.

Throughout histological, molecular, immunological, and biochemical investigations, our data support the idea of an initial (60 days old SOD1-G93A mice) peripheral nerve damage induced by a Wallerian-like degeneration in the development of ALS. We recognize MCs and macrophages as active players in demyelination and inflammatory phenomena involving P/Q calcium channels in NMJ and sciatic nerve. The high and early (in 45 days old SOD1-G93A mice) presence of IgGs in sciatic nerve, the high level of circulating MCs, the activation of Nf-KB pathway in sciatic nerve and the early high level of the cytokines IL-6 and IL-10, allow us to assume that an IgG-dependent and tissue-specific mechanism (35–37), might drive an autoimmune response (38, 39) in SOD1-G93A mice.

## Methods

### Animals

Adult B6SJL-TgN (SOD1-G93A) 1Gur mice (40) expressing high copy number of mutant human SOD1 with a Gly93Ala substitution (SOD1-G93A) originally obtained from the Jackson Laboratories (JAX stock #002726; Bar Harbor, ME, USA) are bred in the animal facility of the Fondazione Santa Lucia, Rome, Italy. The SOD1-G93A progeny has been maintained in the C57BL/6 genetic background by crossbreeding hemizygous SOD1-G93A males with C57BL/6 females to maintain transgene stability as recommended by the Jackson Laboratories guidelines (https://www.jax.org/strain/002726). Screening for the presence of the human transgene was performed as described in Spalloni et al. 2011 (41). Animals were housed in standard transparent plastic cages, in groups of 4 per cage, lined with sawdust under a standard 12/12 h light/dark cycle (7:00 am/7:00 pm), with food and water available ad libitum. The Kaplan-Meyer curves of our male mice (n = 11) using the rotarod test, with the disease onset at 122,5±3,09 days (symptomatic), and the paralyzes at 135±2,51 days, with a body weight decrease from 25 to 23 gr from the onset to the tetraplegia was reported in a recent work (42).

Only male mice were used. They were placed 30’ before sacrifice in experimental room and they were randomly assigned to the different experimental groups. A first investigator performed the surgery and randomly assigned an ID number for organs removal and body fluids. Testing was performed by a blind investigator as for groups for the whole experiment duration. At the end of the statistical analysis the correspondence (ID number/group) was revealed. Care and handling of the animals were in accordance with the guidelines of the European Directive 2010/63/EU, adopted by Council of the European Union for animal experiments, and adequate measures were taken to minimize pain or discomfort. The experimental protocol was approved by the Italian Ministry of Health (Aut. n. 930/2017-PR).

### Animals Age of Samples Collection

All animals sacrifice and tissues collection occurred during presyptomatic stage (no evident signs of disease) at 30–33 days (1 month), at 45–48 days (1.5 month), 60–63 days (2 months), and at disease onset symptomatic stage at 120–123 days (4 months). Hereinafter, to simplify, the animals age will be expressed in months.

### Immunohistochemistry

WT or SOD1-G93A mice of 1, 2, or 4 months old (at least three mice from each experimental group) were sacrificed for immunohistochemistry and perfused with saline followed by 4% paraformaldehyde in phosphate buffer saline (pH 7.4). Sciatic nerves and lumbar spinal cords were collected. Sections (40 μ) of spinal cord tissue and slices (25 μ) of sciatic nerves were prepared as previously described (43).

Tibial muscles were freshly collected and longitudinally positioned on a plate, covered with OCT, placed in methanol for one minute and after in liquid azote. Neuromuscular junctions (NMJ), from 10 μm slices, were stained with: α-bungarotoxin (αBTX) tetramethylrhodamine (TRITC)-conjugated (1:100, Thermofisher); anti-GFAP (glial fibrillary acidic protein, astrocyte marker) antibody (mouse monoclonal, 1:100, Sigma-Aldrich); anti-calcium channel (a1A-subunit PQ) antibody (rabbit polyclonal, 1:100, Sigma-Aldrich); anti-A20 antibody (mouse monoclonal, 1: 500 Santa Cruz Biotechnology).

For double immunofluorescence staining, we used primary and secondary antibodies as in **Table 1**. Nuclei were stained with DAPI (1:1,000, Jackson ImmunoResearch).

**Table 1:** List of primary and secondary antibodies.

PRIMARY	ANTIBODIES			
	SPECIES		PRODUCT	MARKER
NeuN	Mouse monoclonal	1:100	Millipore	Neurons
Cd11b	Rat monoclonal	1:100	Millipore	Microglia/macrophages
Chymase	Goat polyclonal	1:100	Abcam	Mast cells
GFAP	Mouse monoclonal	1:200	Sigma-Aldrich	Schwann cells/Astrocytes
Calcium channel α_1A_Subunit	Rabbit polyclonal	1:100	Sigma-Aldrich	P/Q type Ca^2+^ Channel
ChAT	Goat polyclonal	1:100	Millipore	Acetyltransferase – Cholinergic Neurons
p-p38	Rabbit polyclonal	1:100	Santa Cruz	Phosphorylated p38 (MAP kinase)
NF200	Rabbit polyclonal	1:100	Sigma-Aldrich	Neurofilament 200
P0	Chicken polyclonal	1:200	Millipore	Myelin protein zero
PMP22	Rabbit polyclonal	1:100	Sigma-Aldrich	Peripheral myelin protein 22
CD4	Rabbit polyclonal	1:100	Santa Cruz	T-cells
A20	Mouse monoclonal	1:200	Santa Cruz	key regulatory zinc finger (de)ubiquitinating enzyme A20/tumor necrosis factor α-induced protein 3
**SECONDARY**				
Alexa Fluor 488	Donkey anti- mouse	1:100	Jackson ImmunoResearch	Green
Alexa Fluor 647	Donkey anti-Rabbit	1:100	Jackson ImmunoResearch	Far red
FITC	Goat anti-mouse	1:200	Jackson ImmunoResearch	Green
FITC	Goat anti-rabbit	1:100	Santa Cruz	Green
Cy2	Donkey anti-rat	1:100	Jackson ImmunoResearch	Green
Cy3	Donkey anti-chicken	1:100	Jackson ImmunoResearch	Red
Rhodamine	Goat anti-rabbit	1:100	Jackson ImmunoResearch	Red
HOECHST	bisBenzimide	1:1,000	Sigma-Aldrich	Nuclei

### Immunohistochemistry for the Detection of Immunoglobulins

Cryosections (25 μm) of sciatic nerves derived from WT (2 and 4 months) and SOD1-G93Amice (1, 1,5, 2, 4 months) were incubated for 2 hours with Alexa Fluor 488 anti‐mouse IgG1 (Jackson ImmunoReasearch, 1:200).

### Confocal Microscopy

Images of spinal and nerves immunostained sections were obtained by laser scanning confocal microscopy using a TCS SP5 microscope (Leica Microsystem) while NMJs images were captured by Olympus Fluoview FV1200 (Olympus). All analyses were performed in sequential scanning mode to rule out cross-bleeding between channels. High magnification (40X, 63X) or low magnification (10X) images of spinal cords (lumbar zone L2-L5) and sciatic nerve sections were operated by I.A.S. software (Delta Systems, Italy) while for NMJ (40X) from Olympus confocal images were operated by ImageJ software (version 1.41, National Institutes of Health, USA). Quantification was performed by using the ImageJ. Fluorescence of different proteins observed was quantified (at least 2 slices for each animal) by converting pixels in brightness values using the RGB (red, green and blue) as described in (44).

To confirm double-labelling, confocal Z-stacks were generated. Sections were digitally scanned using 3 channels (488/543/633). For localization of P/Q calcium channels in nerve fibers (magnification 63X zoom 2), 20 frames (stepsize 0.74μm) were acquired from a physical length (z) of 14,08 μm. For immune cell identification in sciatic nerve (magnification 63X zoom 1,40) 17 frames (stepsize 0.49 μm) were acquired from a physical length (z) of 7,90 μm.

### Western Blot (WB)

For the analysis of the P/Q calcium channels expression a total 50 µg of spinal cord samples (L2-L5, lumbar zone) and sciatic nerves, from WT and SOD1-G93A , at two different time points (2 and 4 months) were homogenized in lysis buffer (320 mM sucrose, 10% glycerol, 50 mM NaCl, 50 mM Tris-HCl pH 7.5, 1% Triton X-100, 1 mM PMSF), centrifuged at 4° C for 20 min at 13000 rpm, then supernatants were stored at -20°C. Proteins were applied to sodium dodecyl sulfate–polyacrylamide gel electrophoresis and electroblotted on a nitrocellulose membrane. Membranes were incubated overnight at 4°C with the following primary antibodies: anti-Calcium Channel rabbit polyclonal (α1a Subunit – P/Q-type) (Sigma-Aldrich) (1:500), anti-A20 mouse monoclonal (1: 500) (Santa Cruz Biotechnology), anti- Phospho-NF-κB p65 (Ser536) (93H1, Rabbit mAb #3033, Cell Signaling Technology) (1:1,000), NF-κB p65 (D14E12, XP Rabbit mAb #8242. Cell Signaling Technology) (1:1,000) and β-actin mouse monoclonal (1:1,000) (Abgent). We evaluated the best loading control (**Figure S1**) and confirmed β-actin as housekeeping. Peroxidase-conjugated mouse anti-rabbit IgG (Jackson Immuno Research) (1:40,000) was used as secondary antibody. The βactin bands intensity was used as a control for equal protein loading and measured for densitometric analysis using ImageJ 1.49r software (Wayne Rasband, National Institutes of Health, Bethesda, MA, USA). Each experiment has been repeated 3 times, being each group/experiment composed of 4 sciatic nerves from different animals and 3 lumbar spinal cords.

### Flow Cytometry

Sciatic nerve, spinal cord, and blood derived from different animals of WT or SOD1-G93A mice at two time-points, 2 and 4 months old, were harvested for flow cytometry analysis. Each experiment has been repeated 4 times (n = 3/group/experiment). After having placed the animal’s body on an icy surface, sciatic nerve was removed and collected into HBSS (Hank’s Balanced Salt Solution) with antibiotics (PEN-STREP and gentamicin) that remained on ice until processing. Briefly, sciatic nerve tissues were digested at 37°C in RPMI (with 10% (v/v) fetal bovine serum (FBS) (Sigma-Aldrich), with 40 μl collagenase A (0.8 mg/ml) (Roche Diagnostics) and 100 μl deoxyribonuclease-1 (DNase-1, 400 units/μl) (Sigma-Aldrich). For optimal enzymatic digestion, tissue samples were incubated under slow continuous rotation using a rotator. Following digestion steps, cell suspensions were passed sequentially through 40μm cell strainers (Corning™ sterile cell strainers, Fisher Scientific). Cells were collected from the interface and washed with RPMI at 400 g for 10 min at 20°C. Cells were re-suspended in PBS and kept on ice until proceeding to viability dye staining.

Spinal cord was collected into HBSS (5 ml) with antibiotics (PEN-STREP and gentamicin) that remained on ice until processing. For optimal digestion, tissue sample was crushed and filter (70 μ). Cells were collected from the interface and digested using the same protocol described for sciatic nerves. Finally, cells were re-suspended in HBSS and kept on ice until proceeding to viability dye staining.

Blood was collected in tubes with EDTA. Briefly, 200 μl of blood was diluted in 5 ml of lyse red blood cells (RBCs, 1X water) and left for 5 min at room temperature. After was added 10 ml of PBS. Cells were collected from the interface and washed with RPMI at 400 g for 10 min at 20°C. Cells were re-suspended in PBS (200 μl) and kept on ice until proceeding to viability dye staining.

The cell suspension was stained with the following anti-mouse antibodies: CD45 APC-eFluor 780 conjugated, CD335 PE-eFluor 610 conjugated, and CD3 PE conjugated, all purchased from eBioscience and MHC II Fitc conjugated, F4/80 PE-Vio770 conjugated, CD45R VioBlue conjugated purchased from Miltenyi. To dead cells exclusion we used LIVE/DEAD™ Fixable Aqua Dead Cell Stain Kit purchased from Molecular Probes. For each sample, approximately 300.000 events were selected based on scatter parameters and CD45 positivity, and the analysis was conducted after the exclusion of dead cells and coincident events. The samples were acquired on CytoFLEX Flow Cytometer (Beckman Coulter) and analyzed by FlowJo software.

### Analysis of Serum Cytokines by Luminex Multiplex Cytokine Assay

Serum samples, derived from 2 and 4 months old WT and SOD1-G93A mice (N = 6–11/group), were obtained from blood collected via beheading immediately following euthanasia, allowed to clot at room temperature for 30 min and then centrifuged at 3,000 rpm for 15 min. Serum concentrations of the following soluble factors were measured using a magnetic bead-based 9-plex immunoassay: CCL2/MCP-1, IL-1β; IL-4, CCL3/MIP-1α,IL-10, IL-6, IFN-γ,IL-2, and TNF-α (R&D Systems). Briefly, serum samples were mixed with antibody-linked polystyrene beads on 96-well filter bottom plate and incubate at RT for 2 h on an orbital shaker at 800 rpm. After washing, plate was incubated with biotinylated detection antibody for 1 h T RT. Plate was then washed twice and resuspended in streptavidin-PE. After incubation for 30 min at RT, three additional washes were performed, and the plate was resuspended in reading buffer. Each sample was measured in duplicate along with standards (7-point dilutions) and buffer control. Plates were read using a Luminex Bio-plex 200 system (Life Technologies) for quantitative analysis.

### Statistical Analysis

Experimental groups belong to the same statistical population with similar variance. Immune and biochemical experiments (as indicated in results section) sample size was estimated according to previous experience using the models described.

Experimental data are expressed as mean±SEM. Depending on data, statistical analysis was performed either by unpaired t test or one-way analysis of variance (ANOVA), while for small samples (N < 5 animals) and groups >3 non-parametric analysis was performed by Kruskall-Wallis.

Tukey–Kramer test (parametric analysis) or Dunn Test (non-parametric analysis) have been used for post-hoc analysis in multiple comparison, t-Test for single comparison. Data were considered statistically significant at p <0.05. Statview SAS vers. 5.0, R and GraphPad Prism were used for data analysis.

## Results

### Early Impairment in Peripheral Neurotransmission System: P/Q Calcium Channels Derangement, Myelin Degeneration in SOD1-G93A Presymptomatic Mice and Effects on Spinal Neurons

Since the most relevant sign of ALS disease regards motor functions, we evaluated the health status of the peripheral nerve transmission analyzing nerve endings (NMJ), motor axons (myelinated fibers in sciatic nerves), and motor neurons, in both presymptomatic (2 months) and symptomatic (4 months) SOD1-G93A mice.

**Figure 1:**
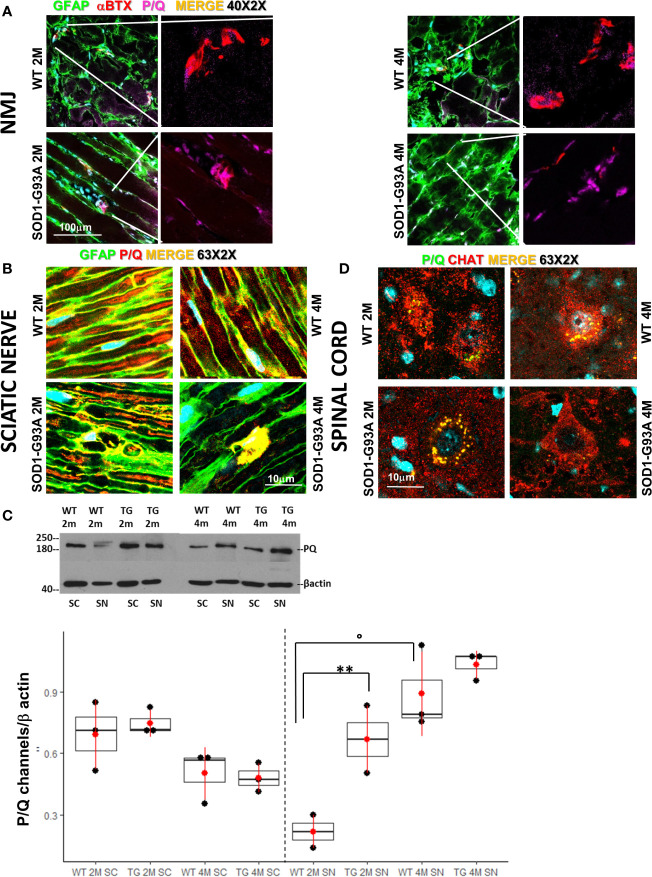
Early degeneration of peripheral P/Q calcium channels in SOD1-G93A mice and effects on spinal neurons. **(A)** Representative examples of confocal images (magnification 40X) of P/Q channels (violet) expression in NMJ (BTX – red) and nerve fibers (GFAP – green) in 2 (2M) and 4 months (4M) WT and SOD1-G93A old mice. **(B)** Sciatic nerves localization and distribution (magnification 63X - zoom 2X) of P/Q calcium channel (red) co-stained with GFAP (green – SCs) in WT and SOD1-G93A mice at 2M and 4M. **(C)** Western Blot analysis of P/Q calcium channels in spinal cord (SC) and sciatic nerve (SN) in WT and SOD1-G93A (transgenic – TG) mice both at 2M and 4M. (**p <0,01 vs WT of the same age; °p <0,05 vs 2M TG ; WB for SN N = 4 each group; WB for SC N = 3 each group; 3 experiments/group). **(D)** Colocalization (magnification 63X zoom 2X) of P/Q calcium channels (green) and CHAT (red) in spinal (L2-L5) motor neurons in WT and SOD1-G93A mice both at 2M and 4M .

We have previously demonstrated the fundamental role played by the P/Q channels to achieve a correct regeneration of injured nerve (34). Indeed, P/Q calcium channels are required for neurotransmission at the NMJ, and are indispensable in SCs physiology (45). In view of these observations we first wondered whether the overexpression of the mutant SOD1-G93A protein alters the number and distribution of the P/Q channels at the NMJ. **Figure 1A** shows P/Q channels normally expressed and equally distributed in the NMJ in 2 and 4 months old WT mice, while in 2 months old SOD1-G93A mice they appear both distributed in the NMJ and condensed in the nucleus. In 4 months transgenic mice, P/Q calcium channel are only localized in the proximity of the nucleus. In the sciatic nerves (**Figure 1B**) of 2 and 4 months old WT mice, the P/Q calcium channels are largely distributed inside the fibers, where they co-localize with GFAP (marker of SC body), and are strongly expressed in Ranvier nodes. In the sciatic nerves of 2 months SOD1-G93A mice immunostaining shows an initial deterioration of the fibers and an aggregation of the P/Q channels in the SCs cytoplasm (delimited by GFAP). At 4 months P/Q calcium channels were strongly localized in the nuclear zone (**Figure 1B** and in z-stack sequences in Video 1). In 1 month old animals, no evident signs of degeneration were present (**Figure S2A**). These observations were supported by the WB analysis (**Figure 1C**), which revealed a greater expression of P/Q channels in sciatic nerves of 2 months old SOD1-G93A mice in respect to the WT (F_1,4_ = 52,467 p = 0.0019), abundance attributable to the accumulation and aggregated status of the proteins, well visible in the IF image (**Figure 1B**). Moreover, as we have already described (34), P/Q calcium channels are differently expressed depending on the age (F_1,4_ =12,915 p = 0.0229). No differences in protein level were appreciable at 4 months.

Both IF and WB analysis were repeated in spinal cords to investigate P/Q calcium channels and the motor neurons status (**Figures 1C, D**). As previously reported (46) and here confirmed, P/Q calcium channels are strongly expressed in motor neurons (**Figure 1D**). No differences in the P/Q channels expression were appreciable between WT and SOD1-G93A mice neither at 2 nor at 4 months (**Figure 1C**). An additional evaluation of motor neurons has been performed analyzing (by means IF) the ChAT expression (**Figure S3**), count of motor neurons and their colocalization withp-p38 (**Figure S4**), confirming (47–51) evident and mild signs of degeneration in 4 months in 2 months old mice, respectively.

**Figure 2:**
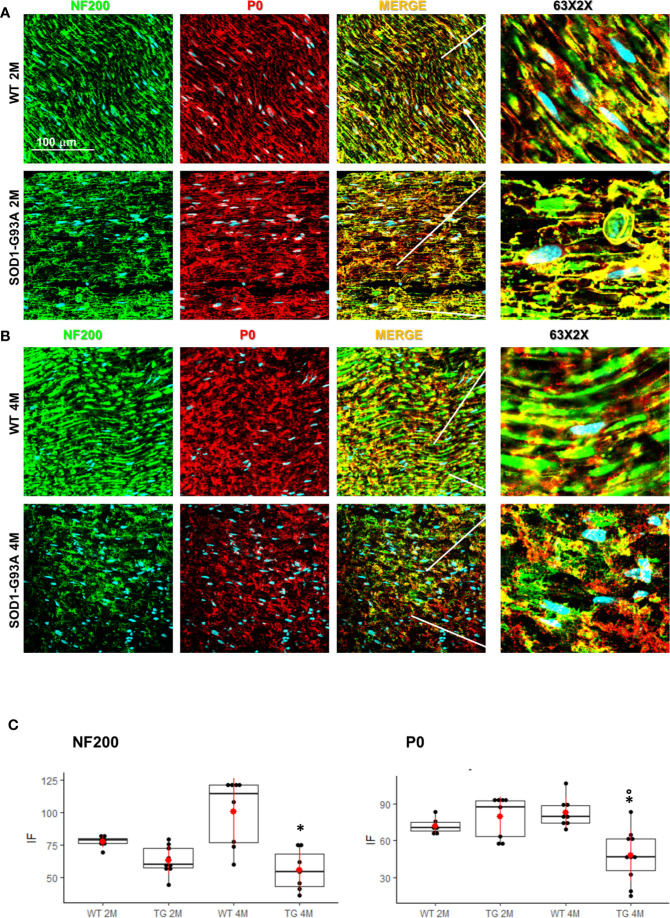
Nerve fibers degeneration in pre- and symptomatic SOD1-G93A mice. **(A)** Representative examples of confocal images (magnification 63x) representing morphological changes in structural (NF200 – green) and myelin organization (P0 – red) in 2 (2M) and **(B)** 4 months old WT and SOD1-G93A mice. The figure shows how myelin protein zero (P0, the major component of the myelin sheath with compacting function) and neurofilament NF200 (NF200, cytoskeletal marker of myelinated fibers) are strongly expressed and widely diffused in WT sciatic nerves. **(C)** RGB analysis of NF200 and P0 in both 2 (2M) and 4 months (4M) old WT and SOD1-G93A (TG) mice. Mean is the red point and +/- SD added as pointrange. (*p <0,05 vs WT; °p <0,05 vs 2M; N = 3/5 group x at least 2 images animal).

**Figure 3:**
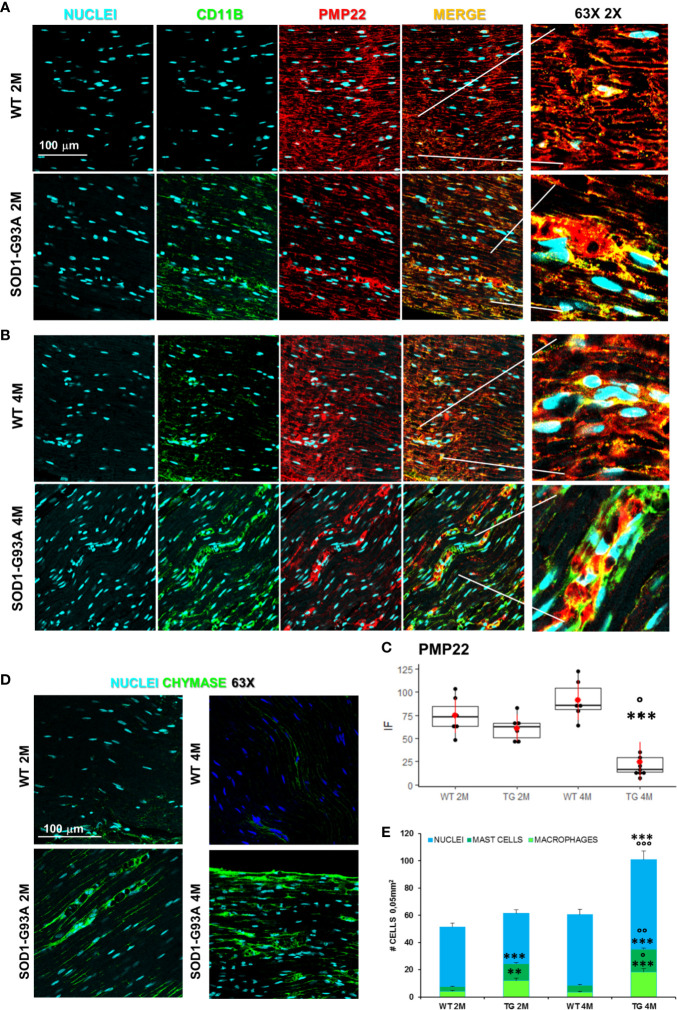
Myelin degeneration and activation of immune-resident cells in SOD1-G93A+ sciatic nerves. **(A)** Representative examples of confocal images of macrophages (green) and PMP22 expression (red) in 2 months and **(B)** 4 months old mice (magnification 63X and zoom 2X). **(C)** RGB evaluation of PMP22 expression in both 2 (2M) and 4 months (4M) old WT and SOD1-G93A (TG) mice. Mean is the red point and +/- SD added as pointrange. (N = 3/5 animals group x at least 2 slices/mouse; °p <0.05 vs 2M and ***p <0.0001 vs WT). **(D)** Sample images (magnification 63X) representing mast cells (green) in sciatic nerves and **(E)** Cells count. Total cells (nuclei, cyan – N = 5–7 animals/group x at least 2 slices/mouse), number of macrophages (light green) and mast cells (dark green) (N = 3–5 animals group x at least 2 slices/mouse). °p < 0.05, °°p < 0,001, °°°p < 0,0001 vs 2M and **p < 0,001, ***p < 0.0001 vs WT.

Motor signal in hindlimbs is transmitted along sciatic nerve and its disruption can consequently generate peripheral and central neuroinflammation as well as locomotor dysfunction. We analyzed structural and myelin proteins. **Figure 2** shows myelin protein zero (P0, the major component of the myelin sheath with compacting function) and neurofilament 200 (NF200, cytoskeletal marker of myelinated fibers) strongly expressed and widely diffused in WT sciatic nerves. From pathomorphological observation, in the 2 months old SOD1-G93A mice (**Figure 2A**) is visible an initial degeneration of neurofilaments and myelin: neurofilaments are fragmented and myelin sheaths are distorted; fibers deconstruction is completed at 4 months (**Figure 2B**), also confirmed by the RGB analysis (NF200: genotype F_1,1_ = 17,671 p = 0.0003; genotype x time F_1,27_ = 6,044 p = 0.02; P0: genotype F_1,1_ = 6,463 p = 0.0168; genotype x time F_1,28_ = 15,217 p = 0.0005) (**Figure 2C**). Normal myelin distribution and conformation was present in 1 month old animals (**Figure S1B**). To further analyze myelin status, another important myelin protein, peripheral myelin protein 22 (PMP22), which plays a role in maintaining myelin integrity, was investigated (**Figures 3A–C**). In 2 months SOD1-G93A old mice, PMP22 presented a high degree of damage characterized by the formation of aggregates, with a decrease of the protein expression (**Figure 3C** – F_1,6_ = 16,357 p = 0.0068) and myelin derangement along all the sciatic nerves (**Figure 3A**). In the nerve fibers of the 4 months old SOD1-G93A mice, PMP22 was irreversibly impaired: it completely disappeared from the fibers, as confirmed by the strong decrease in its expression (**Figure 3C** – F_1,4_ = 38,327 p = 0.0035), and was present only inside macrophages (Cd11b) (**Figure 3B**). In the 1 month old SOD1-G93A mice, the distribution and localization of PMP22 was unchanged (**Figure S1C**).

### Immune Cell-Driven Peripheral Nerve Degeneration at Presymptomatic Stage. 

**Figure 4:**
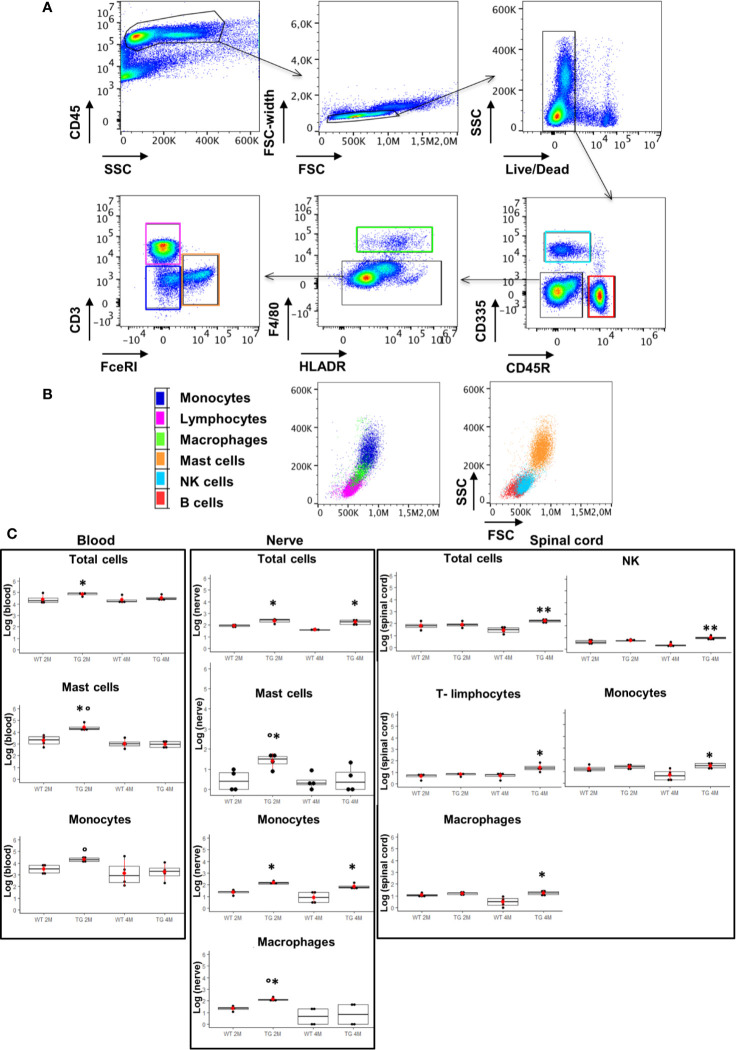
Analysis of CD45 positive cells in blood, sciatic nerve, and spinal cord. **(A)**, **(B)** Identification of hematopoietic origin cells with anti-CD45 antibody; CD45R and CD3 for B and T lymphocytes respectively, CD335 for NK cells, F4/80 and FcεRI for macrophages and mast cells respectively. Results are expressed in absolute number of cells, CytoFLEX Cytometer uses the sample peristaltic pump to take sample and calibrates sample volume delivery for absolute cell counts without using beads. **(C)** Graphs show significant changes (log scale) in circulating immune cells (blood), in sciatic nerve and in spinal cord between wild-type (WT) and SOD1-G93A (TG) mice at the two ages considered ( 2 months – 2M; 4 months – 4M). Mean is the red point and +/- SD added as pointrange. Dunn Test post-hoc, *p < 0.05, **p < 0.001 vs WT; °p < 0.05 vs 4M; 4 experiments/group, each FACS at least 3 different animals.

Since myelin derangement and aggregation is usually attributed, during Wallerian degeneration (WD), to innate immune cells, we examined resident immune cells in sciatic nerves. Macrophages were probed with Cd11b and MCs with Chymase (Chy). The IF analysis (**Figure 3**) shows an impressive early macrophages and MCs activation in 2 months old SOD1-G93A mice, which became devastating at 4 months (**Figures 3A, B, D**), as confirmed by immune cells count (**Figure 3E**; Cd11b: H_3_ = 22,098 p < 0.0001; Chy: H_3_ = 27,549 p < 0.0001). No differences were appreciable in 1 month old SOD1-G93A mice (**Figures S2C, D**), while, as shown in the graph, in 4 months SOD1-G93A mice, considering the elevated total cell number (F_3,58_ = 17,741, p < 0.0001), another cell population could be present (Video 2) together with macrophages and mast cells. To clearly identify the spectrum of immune cells, we performed FACS analysis. We used anti-CD45 antibody to identify all cells of hematopoietic origin, CD45R and CD3 as B and T lymphocytes markers respectively, CD335 for NK cells, F4/80 high expression for monocytes and macrophages, F4/80 low expression together with the physical properties (size-Forward Scatter and granular material inside the cell- Side Scatter) for granulocytes and FcϵRI for mast cells respectively. Results are expressed in absolute number of cells in **Table 2**. In **Figures 4A, B** is showed the gating strategy used to analyze the blood cells, the same analysis was used to identify and characterize CD45 positive cells in spinal cord and sciatic nerve. Since values of immune cells numbers (as shown in **Table 2**) have highly skewed distribution, we used log transformation for data interpretation. In **Figure 4C**, we reported only significantly different cell populations. The total cell number in blood is higher in SOD1-G93A mice than in WT mice during asymptomatic period (2 months - 2M) but not in 4 months (4M) old transgenic mice (H_3_ = 6.86 p = 0.0671; Dunn-Test post-hoc TG 2M vs WT 2M p = 0.0257). This biggest amount of cells is due to the enhancement of circulating MCs (H_3_ = 9.286, p = 0.025; Dunn-Test post-hoc TG 2M vs WT 2M p = 0.04; TG 2M vs TG 4M p = 0.05), an almost significant increase in monocytes (H_3_ = 5.97, p = 0.11; Dunn-Test post-hoc TG 2M vs WT 2M p = 0.06; TG 2M vs TG 4M p = 0.03; TG 2M vs WT 4M p = 0.05) and, although not significant, to the general increase of all immune cells considered (**Table 2**). A significant amount of immune cells is also revealed in sciatic nerves of both 2M and 4M old SOD1-G93A mice (H_3_ =13.09, p = 0.004; Dunn-Test post-hoc TG 2M vs WT 2M p = 0.03; TG 4M vs WT 4M p = 0.007). We found a MCs increase in 2M transgenic mice compared to WT of the same age (H_3_ = 6.485, p = 0.09; Dunn-Test post-hoc TG 2M vs WT 2M p = 0.03) and to 4M old SOD1-G93A mice (Dunn-Test post-hoc TG 2M vs TG 4M p = 0.03), which had a MCs number not significantly different from WT. Moreover macrophages are significantly increased in sciatic nerves of 2M SOD1-G93A mice than in the WT and in 4M SOD1-G93A mice (H_3_ = 9.47, p = 0.02; Dunn-Test post-hoc TG 2M vs WT 2M p = 0.04; TG 2M vs TG 4M p = 0.02); finally a great enhancement of monocytes is found both in 2M and 4M SOD1-G93A mice compared to WT mice at the corresponding age (H_3_ = 12.41, p = 0.006; Dunn-Test post-hoc TG 2M vs WT 2M p = 0.01; TG 4M vs WT 4M p = 0.01). Only during the symptomatic period we found, in spinal cord of SOD1-G93A mice, a significant hematopoietic cells infiltration (H_3_ = 9.55, p = 0.02; Dunn-Test post-hoc TG 4M vs WT 4M p = 0.002), mainly due to T (H3 = 9.43, p = 0.02; Dunn-Test post-hoc TG 4M vs WT 4M p = 0.01) and NK (H_3_ = 11.31, p = 0.01; Dunn-Test post-hoc TG 4M vs WT 4M p = 0.0009) lymphocytes, monocytes (H_3_ = 7.29, p = 0.06; Dunn-Test post-hoc TG 4M vs WT 4M p = 0.01) and macrophages (H_3_ = 9.40, p = 0.02; Dunn-Test post-hoc TG 4M vs WT 4M p = 0.006). The invasion of T-cells in spinal cord is also confirmed by IF analysis as reported in **Figure S5**.

**Table 2:** Immune cells revealed by FACS analysis in blood, nerve, and spinal cord of WT and SOD1-G93A mice at 2 and 4 months.

Facs	BLOOD				NERVE				SPINAL CORD			
	WT 2M	TG 2M	WT 4M	TG 4M	WT 2M	TG 2M	WT 4M	TG 4M	WT 2M	TG 2M	WT 4M	TG 4M
**Total cells**	**17693,75** **SE 2599,76**	**74619** **SE 10947**	**60607** **SE 31097**	**36476** **SE 11612**	**88 ** **SE 11**	**243** **SE 50**	**39 ** **SE 3**	**189** **SE 43**	**79** **SE 29**	**91** **SE 27**	**33** **SE 10**	**174** **SE 28**
**B cells**	**13590** **SE 6763**	**20337** **SE 4306**	**13590** **SE 6763**	**32247** **SE 16570**	**10** **SE 5**	**5** **SE 1**	**3** **SE 1**	**3** **SE 1**	**17** **SE 3**	**15** **SE 3**	**9** **SE 4**	**9** **SE 3**
**T cells**	**13380** **SE 8956**	**16601** **SE 5679**	**11148** **SE 4059**	**9487** **SE 1705**	**1** **SE 0,6**	**6** **SE 3**	**4** **SE 1**	**9 ** **SE 3**	**4** **SE 1**	**5** **SE 1**	**4** **SE 1**	**31** **SE 13**
**NK cells**	**1488** **SE 843**	**2213** **SE 596**	**3188** **SE 1465**	**1638** **SE 484**	**2 ** **SE 1**	**4** **SE 0,7**	**1 ** **SE 0,4**	**4** **SE 1**	**3** **SE 1**	**4 ** **SE 0,5**	**1** **SE 0,5**	**8** **SE 1**
**Macrophages**	**3761** **SE 1760**	**18874** **SE 7594**	**10954** **SE 9908**	**3936** **SE 2600**	**24** **SE 5**	**127** **SE 43**	**12** **SE 6**	**78** **SE 24**	**11** **SE 2**	**15** **SE 2**	**3** **SE 1**	**15** **SE 2**
**Monocytes**	**4065** **SE 1754,2**	**21561,25** **SE 5855,39**	**10954** **SE 9908**	**4064** **SE 1754,2**	**1** **SE 0,6**	**14** **SE 8**	**2** **SE 0,5**	**54** **SE 35**	**7** **SE 4**	**10** **SE 3**	**3** **SE 2**	**16** **SE 7**
**Mast cells**	**2636** **SE 1253**	**9639** **SE 3860**	**1421** **SE 236**	**2225** **SE 1293**	**3** **SE 2**	**15** **SE 13**	**2** **SE 1**	**6 ** **SE 5**	**0**	**0**	**1**	**5 ** **SE 4**
**Others**	**1007** **SE 695**	**6868** **SE 5197**	**1421** **SE 236**	**2225** **SE 1293**	**45** **SE 10**	**76** **SE 14**	**13** **SE 4**	**76** **SE 31**	**36** **SE 20**	**39** **SE 21**	**9** **SE 2**	**80** **SE 11**

The table resumes the mean value (SE = standard error) of different immune cells. Red values indicate the significant differences between genotypes (TG = transgenic mice - SOD1-G93A mice vs WT = wild type mice) while green value are the significant differences regarding the age (2M vs 4M).

**Figure 5:**
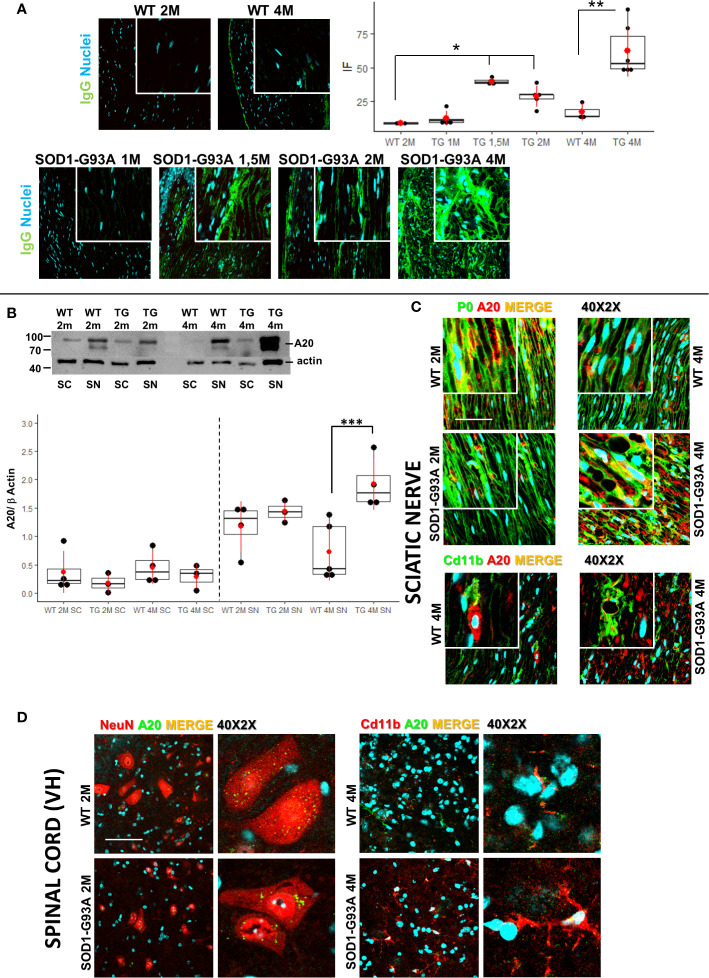
Early detection of immunoglobulins and A20 expression. **(A)** IgGs detection (green) in sciatic nerves of 2 (2M) and 4 months (4M) WT mice compared with the IgGs presence in 1, 1,5, 2, and 4 months old SOD1-G93A mice. Dunn-Test post-hoc * vs WT 2M p <0.05; ** vs WT 4M p <0.01. **(B)** Western blot analysis of A20 levels in sciatic nerve (SN) and spinal cord (SC) in 2 (2M) and 4 months (4M) old WT and SOD1-G93A (TG) mice. Dunn Test post-hoc ***vs WT 4M p <0.00; 3–4 experiments/group, each WB at least 3 different animals. **(C)** Confocal sample images of A20 (red) localization and distribution co-stained with myelin protein P0 (green) or macrophages (Cd11b – green) in sciatic nerves of 2 or 4 months old WT and SOD1-G93A mice. **(D)** Confocal sample images of A20 (green) distribution and localization co-stained with neurons (NeuN – red) or microglia (Cd11b – red) in spinal cord of 2 or 4 months old WT and SOD1-G93A mice.

We examined some characteristic signs of immune-mediated diseases to corroborate the hypothesis that early inflammatory phenomena could be at the origin of ALS axonopathy. Since myelin derangement and aggregation is one of the first occurrence in SOD1-G93A, we decided to detect autoantibodies (IgGs) in sciatic nerves as first evidence of an immunological disorder. As shown in **Figure 5A** immunofluorescent IgGs are almost absent in sciatic nerves of WT mice at 2M and 4M as well as in 1 month old transgenic mice while a significant increase in fluorescence is already appreciable in 1-month and half old SOD1-G93A mice, increase that is substantially maintained in 2 months and strongly enhanced in 4 months old mice (H_5_ = 21.8, p = 0.00056; Dunn-Test post-hoc TG 1.5M vs WT 2M p = 0.017; TG 2M vs WT 2M p = 0.014; TG 4M vs WT 4M p = 0.013).

Since NF/kB pathway controls innate and adaptive immune response and the protein A20 is a master regulator of NF/kB (46), we decided to evaluate A20 levels and localization (**Figures 5B–D**) in sciatic nerves and spinal cords both in 2 and 4 months old mice. Western blot analysis (**Figure 5B**) did not evidence any difference in A20 level both in sciatic nerves and spinal cords of 2 months animals, on the contrary, in 4 months old mice, while in spinal cords there weren’t any appreciable differences between genotypes, in sciatic nerves a strong increase in A20 level is detectable (H_3_ = 10.6 p = 0.01; Dunn Test post-hoc TG 4M SN vs WT 4M SN p = 0.001). To evaluate activation of NF/kB, we performed WB in spinal cords of both 2 and 4 months old mice. Densitometry did not reveal any appreciable significance in all groups analyzed (**Figure S6**), supporting the data on a peripheral immune-mediated response.

To corroborate WB data, we performed A20 immunostaining in all tissues and ages considered. As shown in **Figure 5C**, IF images confirm a similar distribution and localization of A20 in sciatic nerves of 2 months old WT and SOD1-G93A mice, while it is particularly evident in 4 months old transgenic mice a strong presence of A20, which, from a morphological point of view, appears in aggregate form inside myelinated fibers (P0) or partially expressed in macrophages (Cd11b).

Also, immunostaining of spinal cords confirmed WB analysis, since A20 results not differently distributed and expressed in 2 and 4 months old mice. Moreover, we have co-stained A20 with NeuN (neurons) or Cd11b (microglia) to evaluate possible different expression in different cell population. Confocal images revealed an A20 constitutive presence in neurons and microglia and as expected a microglia activation (different morphology) in 4 months old SOD1-G93A mice.

**Figure 6:**
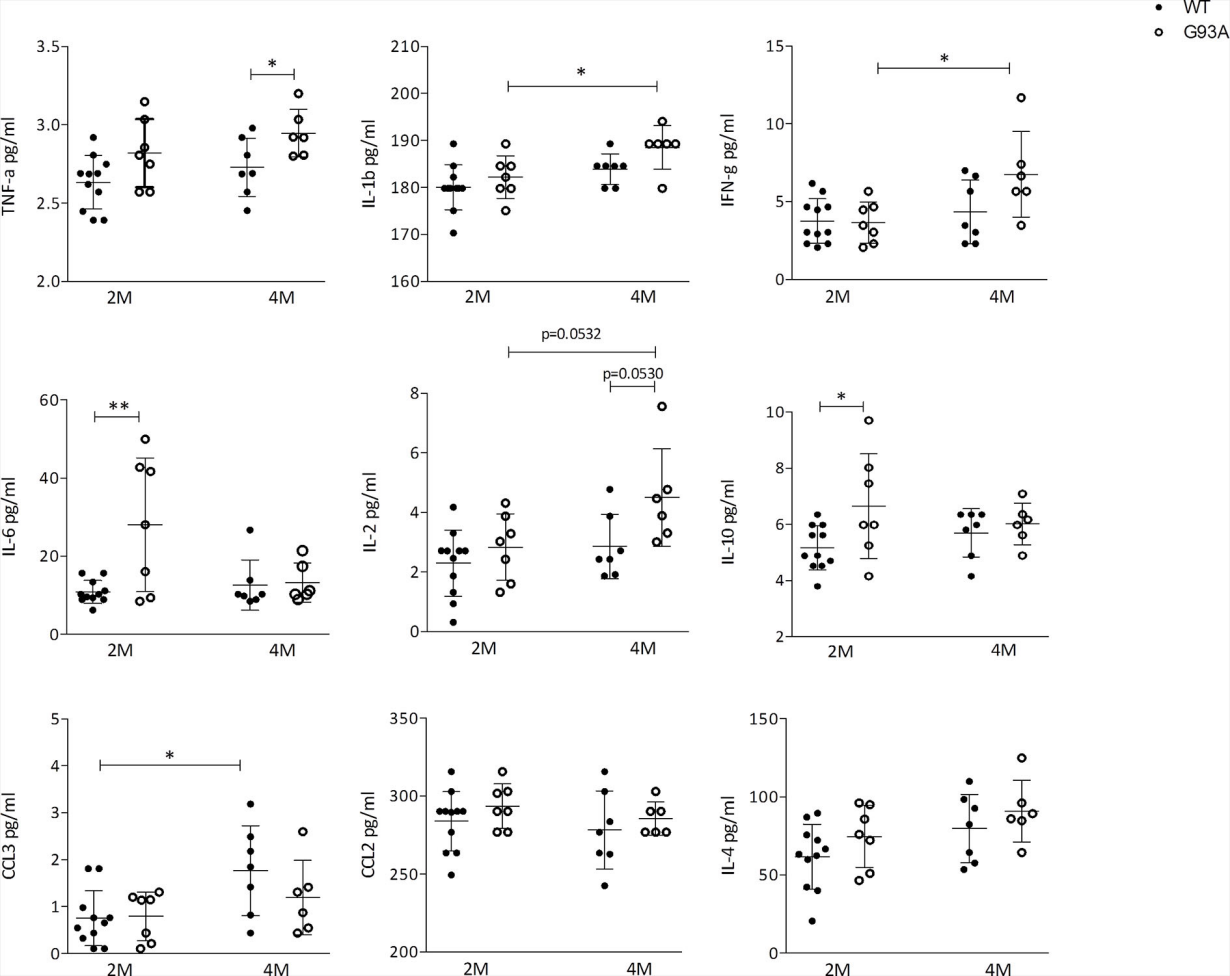
Cytokines production in WT and G93A mice serum. Graphs showing the level of: TNF-α IL-1β; IFN-γ; IL-6; IL-2; IL-10; CCL3/MIP-1αCCL2/MCP-1 and IL-4 in serum of 2 months (n = 11) and 4 months (n = 7) WT mice and 2 months (n = 7) and 4 months (n = 6) SOD1-G93A+ mice. p values are calculated with unpaired t-test: *p < 0.05; **p < 0.01.

Finally, to reveal biomarkers of inflammation, to confirm the immunological response and to support cellular data, we quantified the circulating cytokines in serum of SOD1-G93A mice by means of LUMINEX. Pro-inflammatory agents were found increased in transgenic mice compared to WT. In particular, TNF-α levels were significantly higher (p = 0.0435) in SOD1-G93A mice compared to WT mice at 4 months (**Figure 6**). IL-1β as well as IFN-γconcentration (p = 0.0309; and p = 0.0226; respectively) and the number of cell total amount increased significantly from 2 to 4 months in SOD1-G93A mice but not in WT. Moreover, the serum level of IL-6 concentration was significantly higher (p = 0.0044) in 2 months old SOD1-G93A mice compared to WT mice at the same age. At different time frame other non- inflammatory cytokines arose. Levels of IL-2 showed a tendency to increase (p = 0.0532) from 2 to 4 months in SOD1-G93A mice as well as between the two genotypes (p = 0.0530). IL-10 concentration was significantly higher (p = 0.0318) in the SOD1-G93A mice compared to the WT mice, at 2 months. Finally, WT mice showed a CCL3 concentration significantly increased (p = 0.0128) from 2 to 4 months. Other cytokines investigated (CCL2 and IL-4) did not differ between the groups and time frame considered.

### Central Neuroinflammation During Peripheral Disease Progression.

**Figure 7:**
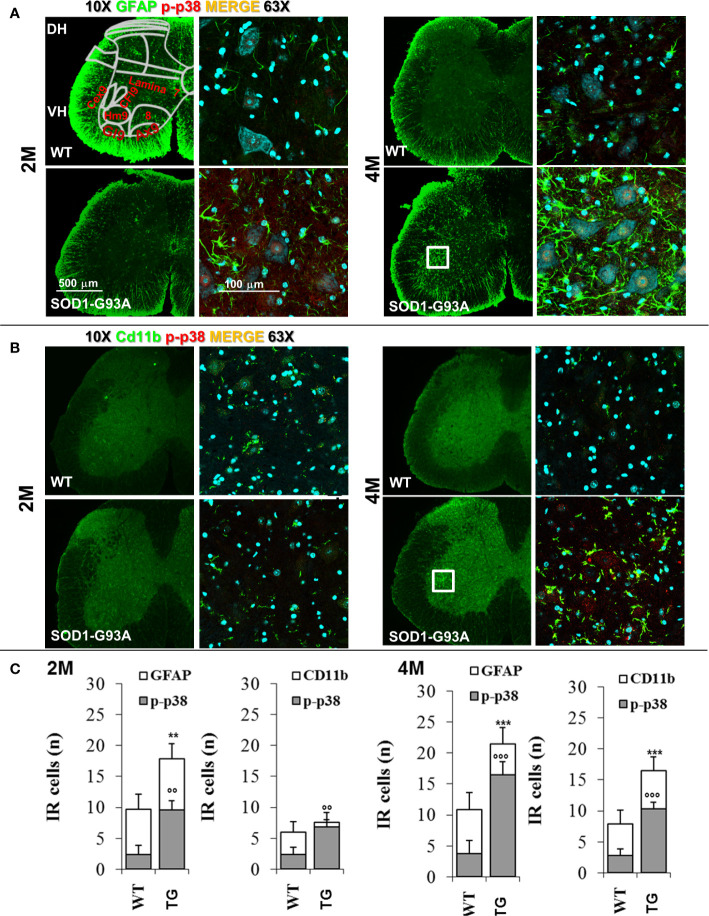
Glia activation in SOD1-G93A+ mice. **(A)** Representative examples of confocal images of spinal astrocytes (GFAP – green) and their activation (colocalization with p-p38 – red) in lumbar sections (magnification 10x and 63x) of 2M and 4M WT and SOD1-G93A+ mice and **(B)** microglia (Cd11b – green) activation (p-p38 – red). Schematic representation of ventral laminae (VH – ventral horn, lamina 9: Cex9 – crual extensor, CFl9 – crural flexor, Hm9 - harmistring, Gl9 – gluteal muscle, Ax9 – axial muscle, 8 – lamina 8) is shown in first panel. **(C)** Immune-responsive cell number for GFAP and Cd11b colocalizion for p-p38. **p < 0.01 ***p < 0.001 vs GFAP or CD11b WT; °°p < 0.01 °°°p < 0.001 vs p-p38 WT.

To support results of primary and early peripheral inflammation and neurodegeneration, we were looking for signs of neuroinflammation and degeneration at the central level. We have evaluated the timing of spinal astrocytes reaction and microglia activation. As **Figure 7** shows, in 2 months old SOD1-G93A mice a beginning of microglia (Cd11b - t_16_ = 3.126; p = 0.0065) and astrocytes activation (GFAP - t_16_ = 7.222; p = 0.0006) is appreciable by the colocalization with p-p38, other than an increase in the astrocytes number (t_16_ = 3.149; p = 0.0062) in comparison with WT, not revealed in asymptomatic SOD1-G93A mice (1 months and half old, **Figure S7**). In 4 months old animals microglia and astrocytes were hyperreactive (t_16_ = 6.362; p < 0.0001 t_16_ = 5.635; p < 0.0001, respectively) especially around the motor neurons of the ventral area, which is associated to hindlimb motor control (lamina IX) such as hamstring, gluteal muscles, and crual extensor (HM9, Gl9, CEx9); this hypperactivation coincides with the period of total sciatic nerve demyelination in SOD1-G93A mice.

## Discussion

In the last few years different studies (47, 48) have highlighted the possibility that in ALS the motor neurons disease can be caused by a distal axonopathy and that the immune system plays a role in peripheral neurodegeneration (15, 19). Wallerian degeneration (52) is a process of axons degeneration starting after nerve injury and consisting of different steps. When it starts lysosomial and oxidative activity enhances in SCs. In particular, neurofilaments, neurotubules, myelin, and axons are fragmented. Concomitantly, resident mast cells and macrophages are activated, to promote inflammation and immune reaction (52) the first and to engulf myelin and axons debris the second. According with different studies (53–55), we have shown in SOD1-G93A mice an early Wallerian-like degeneration that precedes the central degeneration. A very initial immune activity was evidenced by the presence of IgGs on sciatic nerve in 45 days old mice followed in 60 days old mice by myelin and nerve fibers degeneration, strong presence of IgGs, MCs and macrophages in sciatic nerve, circulating MCs, macrophages, IL6, and IL10.

Moreover, since a role of P/Q calcium channels in ALS development has been suggested (28, 29) we have also investigated the P/Q calcium channels in both NMJ and sciatic nerve associated to SCs. These channels are responsible for the release of neurotransmitters and for health and proper development of SCs (33, 34, 45, 46); a defect in their expression can cause important demyelinating disease and motor dysfunction (30–33, 56). Moreover, some researches have referred the P/Q calcium channel as potential immunological target in ALS (27, 29). In line with these hints, our results, for the first time, have revealed a severe impairment in the expression and tissue distribution of the P/Q calcium in the SCs: in 2 months old SOD1-G93A mice a status of P/Q aggregation inside SCs was found, while a nuclear localization was visible within SCs in 4 months old mice. Moreover, the WB analysis revealed an upregulation of these channels in 2 months transgenic animals as though a compensatory mechanism occurred. There is scarce literature on this topic, Takahashi et al. (57) uncovered that in spinocerebellar ataxia type 6 the aggregation of α1A voltage-gated calcium channel C-terminal fragment (Cav2.1-CTF) in the cytoplasm can cause cell death. P/Q calcium channels are also expressed in spinal motor neurons (46) and our WB results show that these channels were not critically altered in respect to the WT animals, at both ages analyzed, reinforcing our hypothesis that an initial degeneration moves from the peripheral nerve and is followed by the spinal involvement.

To define the role of immune system in myelin and P/Q degeneration, we simultaneously investigated, by means of immunohistochemical/confocal and Flow cytometry (FCM) analysis, in sciatic nerves, spinal cord and blood, the resident, invading and circulating immune cells. Our analyses (FCM and IF) of the SOD1-G93A mice sciatic nerve specimens at 2 months, have revealed a massive presence of macrophages and mast cells in sciatic nerves, as well as a strong increase in circulating monocytes and mast cells, while no alterations in 2 months old SOD1-G93A mice spinal cords were observed. When 4 months SOD1-G93A mice were investigated, nerves appeared definitively compromised with a massive presence of macrophages, monocytes and other not identified cells; on the other hand, in blood no differences were appreciable in the distribution and amount of the immune cells in respect to WT. Finally, immune infiltration (T-cells, NK cells as well as macrophages and other cells) was observable in spinal cord of 4 months old SOD1-G93A mice. Therefore, we have discovered a real and early immune reaction characterized by a progressive cell invasion that during the symptomatic period, in line with a previous study (18, 55) revealed the devastating presence of macrophages in sciatic nerves. It is peculiar, in fact, the evidence of a high rate of systemic mast cells in the presymptomatic mice. Normally, mast cells are scarcely represented in blood and when they are found, they are linked to an allergic reaction or an autoimmune disease (39). Trias and colleagues (58) have reported the presence of degranulated mast-cells in the NMJ of symptomatic SOD1-G93A rats, suggesting a likely role in ALS axonopathy.

Mast cells are crucial effector cells in many settings of the immune response, including host defense, immune regulation, allergy, chronic inflammation, and autoimmune diseases. The idea that MCs are involved in the initiation and sustaining events of autoimmunity is based on abundant data from studies of both human disease and animal models (59). They are involved in the initiation and in both the induction and effector phases of adaptive immunity (27, 39). Mast cells can be activated by a plethora of stimuli such as pro-inflammatory agents and IgGs able to induce MCs degranulation, release of histamine, cytokines and chemokines, proliferation and migration (35). In fact, it has become clear that MCs play a fundamental role in IgG-dependent tissue-specific autoimmune diseases (36). For that reason, we have analyzed the presence of IgGs on sciatic nerve, since myelin and P/Q calcium channels degeneration appear to be the first evidence in SOD1-G93A mutation-induced axonopathy. Surprisingly, we found a significant IgGs increase already in asymptomatic mice (1 month and half old) persisting in 2 and 4 months old SOD1-G93A mice.

Regulation of inflammation and immune response is mediated by NF-kB signalling that, when altered, is linked to autoimmune and inflammatory disease. A control function of this system is attributed to A20 protein (tumor necrosis factor alpha-induced protein 3, TNFAIP3). A20 is induced by the NF-kB canonical pathway and is well known to inhibit it, thus exerting repression of inflammatory phenomena (60). However, some works reported that when overexpressed, A20 might also induce the activation of NF-kB non-canonical pathway (61). According with this view, we can assume that the A20 upregulation observed in sciatic nerve of 4 months old SOD1-G93A mice can be ascribed to an activation of the non-canonical pathway, fitting with the idea that A20 (62), when aberrantly activated, in different cell types, can promote autoimmunity and inflammation.

Our data that indicate monocytes, mast-cells, and macrophages as early players in the ALS pathophysiology, are further corroborated by the identification of pro-inflammatory mediators. We found an upregulation of IL-6 and IL-10 in blood of 2 months old SOD1-G93A mice in comparison with values derived from WT mice. Both interleukins can be considered myokines (63), but they are also produced and released by mast-cells in response to allergy or in autoimmune diseases (64–66).

Moreover, we have recently demonstrated, in different animal models of denervation, including the SOD1-G93A mice, that the STAT3-IL6 signaling is activated in muscle resident fibro/adipogenic progenitors and promotes myofibers atrophy and fibrosis (67). Clinical studies also (68, 69) support the idea that IL-6 and mast-cells could be key factors in the comprehension of the ALS pathogenesis. Finally, histaminergic system is dysregulated in ALS animals (70) and recent work demonstrated that treatment of SOD1-G93A mice with the antihistaminergic drug clemastine is able to reduce microgliosis, to enhance motor neuron survival (71), and to improve neurological symptoms (72), thus supporting the idea of a role for mast cells in the very early phases of ALS.

In the late phase (4 months), in SOD1-G93A mice we found an increased expression of the cytokine TNFα, which is mainly released by macrophages and considered a pro-inflammatory and apoptotic cytokine, and of IL-2, which is produced by T-cells to limit autoimmune and allergic reaction. The increase level of TNFα, together with overexpression of A20, reinforces the hypothesis that NF-kB non canonical pathway can be activated during ALS progression (73).

Finally, we found that other chemokines and cytokines (IFNg, CCL3 and IL-1b) were enhanced in 4 months old compared to 2 months old SOD1-G93A mice; it is noteworthy that they are all linked to macrophages attraction, recruitment and activation.

Lastly, to conclude and monitor the progression of ALS signs from the peripheral nerve to the spinal cord, we investigated neuroinflammation timing. In 2 months old familial ALS mice we observed a bland astrocytic reaction (not present in animals of 1 month and half old) and the absence of microglia activation; on the contrary, hyperreactive astrocytes and activated microglia were present in 4 months animals, particularly in the ventral horn areas around motor neurons which control the sciatic nerve—single muscle communication such as HM9, Gl9, and CEx9. Although autoimmunity in ALS isn’t a novel concept (27), little is known about the early events that trigger immune response. We depict a hypothesis focused on MCs initiation and propagation of autoimmune disease that could be similar to what happen in Multiple sclerosis (MS) (74). Briefly, in MS, MCs throughout cytokines/chemokines release recruit and activate T cell/macrophage after MCs present myelin antigen to T cell, disrupt the BBB to allow the infiltration of activated T cells into the brain and target in myelin basic protein. Moreover, MCs damage myelin enhancing demyelination and inflammation (59, 75–77). Similarly, we propose that, differently from MS, MCs have a role in early degeneration in peripheral nervous system, affecting myelin and Schwann cells functions and having a ripple effect on neuronal damage, an hypothesis consistent with the evidence that ALS is a motor neuron pathology that in SOD1 ALS mouse model as well as in ALS patients affects first distal axons (4, 78, 79).

In conclusion, our research supports the recent notion that mast-cells and its modulators (IL-6, IL-10 and IL-2) can be drugable targets in ALS and that compounds able to alter mast-cells pathogenic effects should be tested in different ALS murine models (80).

## Data Availability Statement

The raw data supporting the conclusions of this article will be made available by the authors, without undue reservation.

## Ethics Statement

The experimental protocol was approved by the Italian Ministry of Health (Aut. n. 930/2017-PR).

## Author Contributions

Conceptualization, SM. Data curation, SM, DA, FD, VV. Formal analysis, SM, LB, FP, PL. Investigation, SM, FD, DFA, EP, MN, AS, CP, FrP. Methodology, SM, FD, DFA. Supervision, SM, FP, LB, PL. Writing—original draft, SM. Writing—review and editing, FP, LB, DFA, PL, AS, FD, VV. All authors contributed to the article and approved the submitted version.

## Conflict of Interest

The authors declare that the research was conducted in the absence of any commercial or financial relationships that could be construed as a potential conflict of interest.
