# Corrigendum: Very Early Involvement of Innate Immunity in Peripheral Nerve Degeneration in SOD1-G93A Mice

**DOI:** 10.3389/fimmu.2021.682508

**Published:** 2021-04-06

**Authors:** Daniela Francesca Angelini, Federica De Angelis, Valentina Vacca, Eleonora Piras, Chiara Parisi, Michele Nutini, Alida Spalloni, Francesca Pagano, Patrizia Longone, Luca Battistini, Flaminia Pavone, Sara Marinelli

**Affiliations:** ^1^ Neuroimmunology Unit, IRCCS Santa Lucia Foundation, Rome, Italy; ^2^ CNR—National Research Council, Institute of Biochemistry and Cell Biology, Rome, Italy

**Keywords:** Peripheral nerve degeneration, demyelination, amyotrophic lateral sclerosis, mast cells, monocytes/macrophages, pro-inflammatory cytokine, autoimmunity

In the published article, there was an error regarding the equal contributions label (‡). Valentina Vacca should also have this label.

The authors apologize for this error and state that this does not change the scientific conclusions of the article in any way. The original article has been updated.

