# Supplementary material for: Very Early Involvement of Innate Immunity in Peripheral Nerve Degeneration in SOD1-G93A Mice
Source: Front Immunol. 2020 Nov 20;11:575792. doi: 10.3389/fimmu.2020.575792 (PMC7714949; doi:10.3389/fimmu.2020.575792)
Supplement: Supplementary file 3 [file DataSheet_1.docx]

Supplementary Material

**Figure S1**


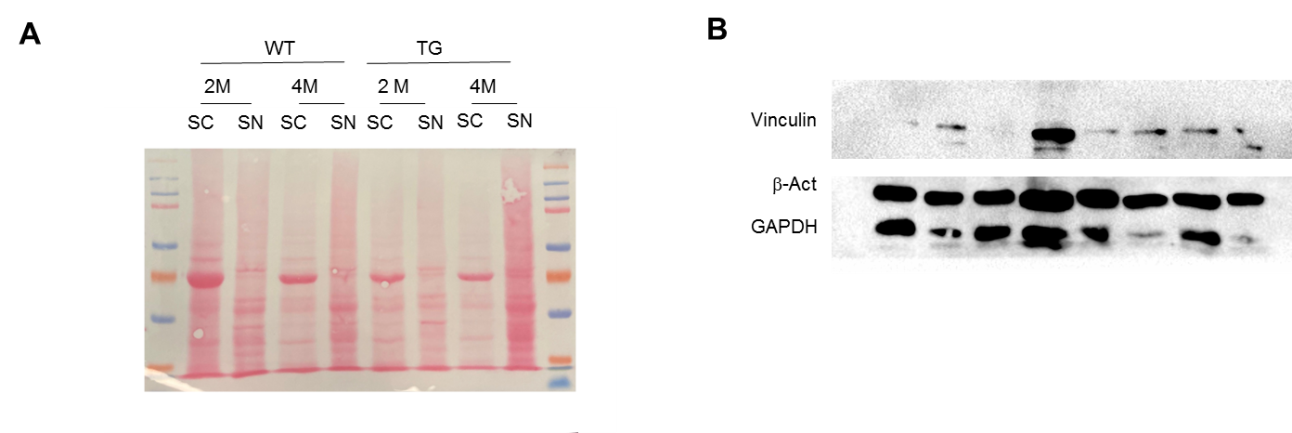


Supplementary Figure 1. Evaluation of total control protein loading. A) Ponceau S staining of total protein. WT=wild type; TG= SOD1-G93A mice; 2M=2 months; 4M=4 months; SC=spinal cord; SN=sciatic nerve. B) Three different largely used loading controls (GAPDH, VINCULIN and β-actin), were tested and β-actin was selected because showed the most stable expression between samples.

Figure S2
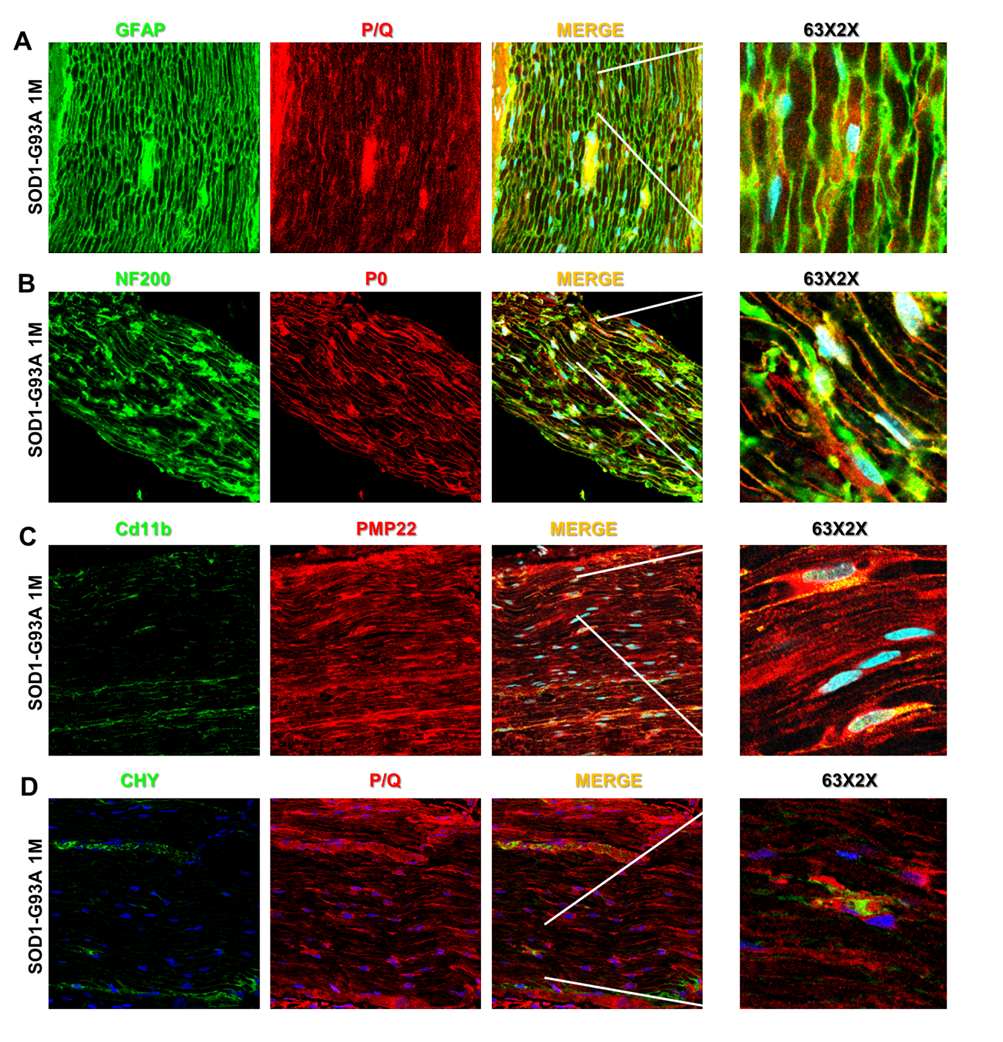
Supplementary Figure 2. Sciatic nerve of 1 month old SOD1-G93A mouse. A) P/Q calcium channels (red) are largely distributed inside the fibers, where co-localize (yellow) with GFAP (green- marker of SC body), no sign of degeneration is observable. Normal myelin B) (P0 - red) and C) (PMP22 - red) distribution and conformation is present in 1 month animals as well as no structural defects are appreciable (NF200 - green). No differences in C) macrophages (Cd11b – green) or D) mast cells (CHY – green) are appreciable in 1 month old SOD1-G93A mice.

**Figure S3**

*
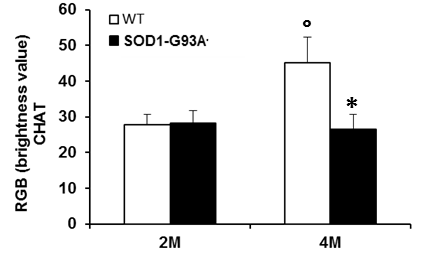
*

**Supplementary Figure 3.** ChAT expression in spinal motor neurons (L2 – L5). RGB evaluation (*p<0,05 vs 4m WT; °p<0.05 vs 2M WT; N=3 group) of ChAT in lumbar spinal cord of both WT and SOD1-G93A at 2 and 4 months (2M and 4M). ChAT expression was found increased in 4 months WT in respect of 2 months old mice, probably due to motor system maturation. This difference was not recognizable in mutant mice (between 2 and 4 months) and determined the significant reduction that we observed, when compared with 4 months old WT mice (H_3_=10,448; p=0.0151; Dunn-Test post-hoc WT 2M vs WT 4M p=0.0053; WT 4M vs TG 4M p=0.006) indicating a motor neurons sufferance.

**Figure S4**


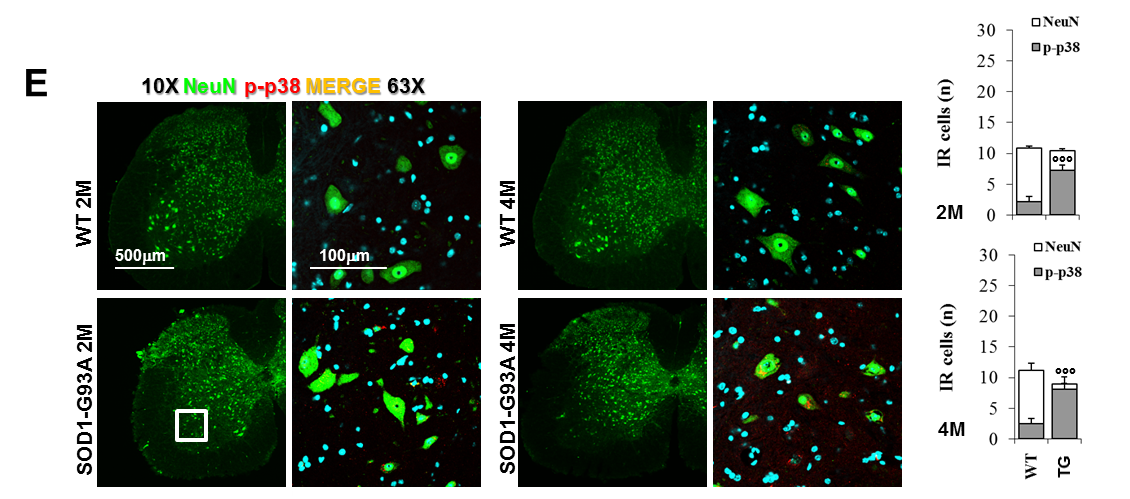


**Supplementary Figure 4**. Colocalization of neurons (green) and p-p38 (red) staining for activation. Low magnification (10X) and high magnification (63X) of lumbar sections of WT and SOD1-G93A spinal cords (from 2M and 4M old mice) are represented and immune-responsive (IR) cells in ventral horns were counted (SOD1-G93A mice = TG, transgenic) °°°p<0,0001 vs p-p38 WT; N=3 group).

The p-p38 staining showed a hyperactivation (t_16_=5.167; p<0.0001) in 2 months SOD1-G93A motor neurons, exacerbated at 4 months (NeuN/p-p38 colocalization).

**Figure S5**


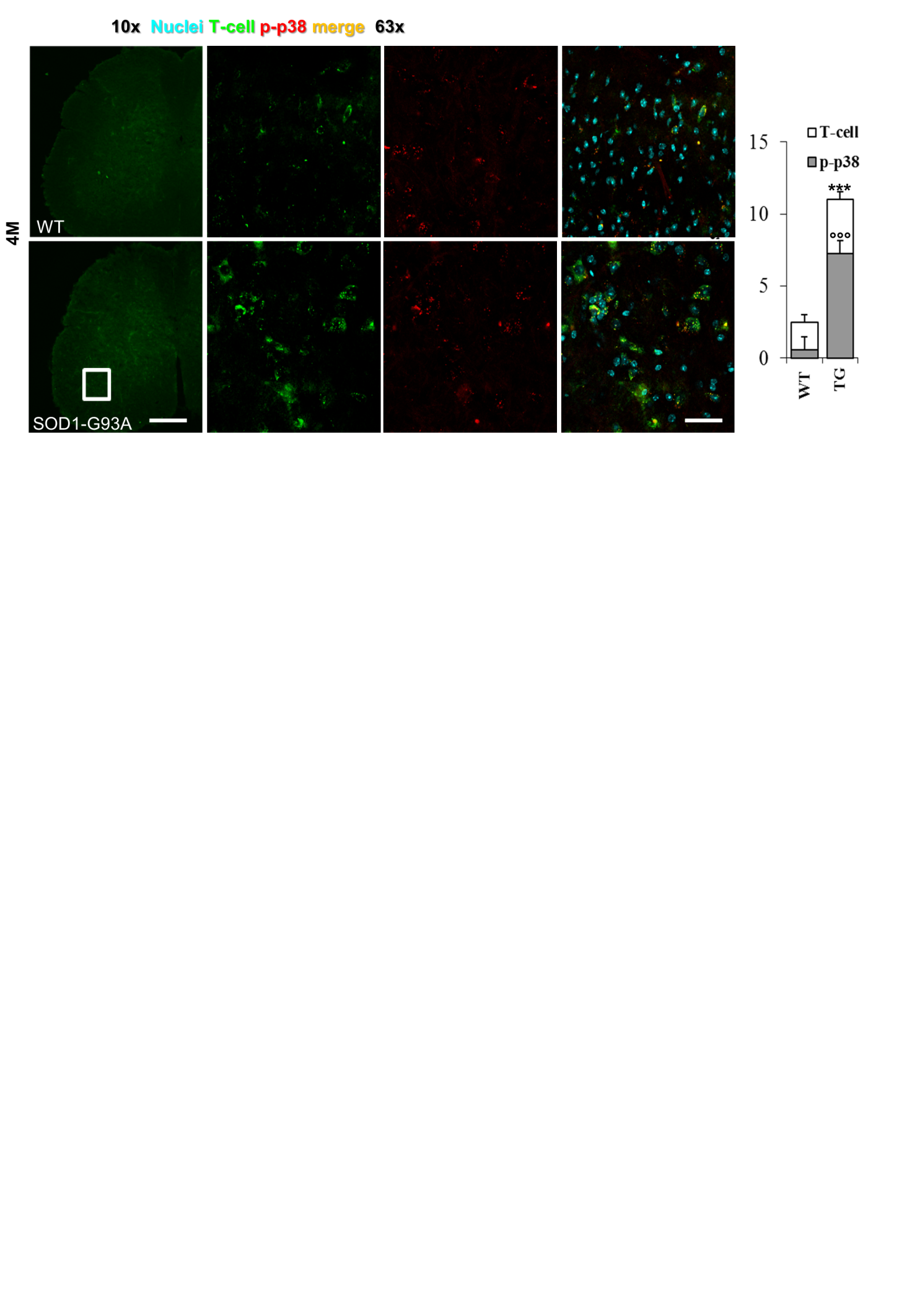


**Supplementary Figure 5.** **Immunofluorecence analysis of T-cells in spinal cord.** Co-staining of T-cells (green) and p-p38 (red) in spinal cord of 4M WT and SOD1-G93A mice. Graph shows total T-cells count (white) and T-cells expressing p-p38 (grey) in spinal ventral horn. ***p<0,0001 Total T-cell vs WT (t_16_=7,216); °°° p<0,0001 T-cell expressing p-p38 vs WT (t_16_=7,674).

**Figure S6**

**
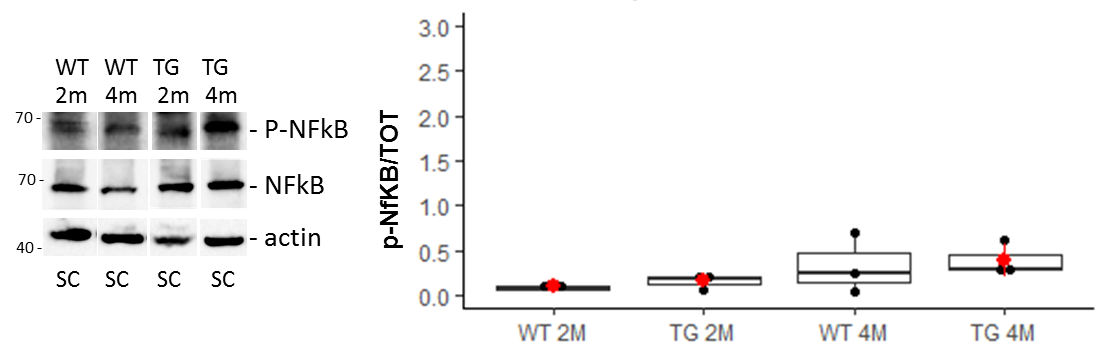
**

**Supplementary Figure 6**. Western Blot analysis for NFkB phosphorilation in spinal cord. A) Representative western blot image showing the phosphorilated (-P) and total NFkB protein expression in mouse spinal cord samples (SC) isolated from 2 and 4 months old WT and transgenic (TG) mice . Beta actin was used as loading control. B) Box-plot showing the densitometric analysis of P–NFkB/NFkB bands intesity ratio. .

**Figure S7**


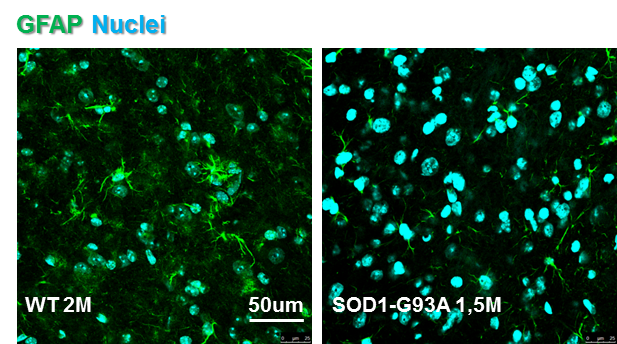


**Supplementary Figure 5.** **Immunustaining of astrocytes in spinal cord of asymptomatic mice**. Evaluation of astrocytosis (GFAP – green) in spinal cord of 2 months (2M) old wild-type (WT) and 1 month and half old (1.5M) SOD1-G93A mice.
